# Improving Lung Cancer Screening Selection: A Comparative Analysis of Risk Models and Traditional Criteria in a Western European General Population

**DOI:** 10.3390/cancers18050724

**Published:** 2026-02-24

**Authors:** Danrong Zhong, Grigory Sidorenkov, Marcel J. W. Greuter, Colin Jacobs, Pim A. de Jong, Hester A. Gietema, Harry J. M. Groen, Firdaus A. A. Mohamed Hoesein, Noa Antonissen, Ralph Stadhouders, Harriet L. Lancaster, Marjolein A. Heuvelmans, Rozemarijn Vliegenthart, Geertruida H. de Bock

**Affiliations:** 1 Department of Epidemiology, University Medical Center Groningen, University of Groningen, 9713 GZ Groningen, The Netherlands; d.zhong@umcg.nl (D.Z.); g.sidorenkov@umcg.nl (G.S.);; 2 Department of Radiology, University Medical Central Groningen, University of Groningen, 9713 GZ Groningen, The Netherlands; m.j.w.greuter@umcg.nl (M.J.W.G.);; 3 Diagnostic Image Analysis Group, Department of Medical Imaging, Radboud University Medical Center, 6525 GA Nijmegen, The Netherlands; colin.jacobs@radboudumc.nl (C.J.);; 4 Department of Radiology, University Medical Center Utrecht, 3584 CX Utrecht, The Netherlands; p.dejong-8@umcutrecht.nl (P.A.d.J.);; 5 Department of Radiology and Nuclear Medicine, Maastricht University Medical Center, Maastricht University, 6229 HX Maastricht, The Netherlands; 6 GROW School for Oncology and Reproduction, Maastricht University, 6229 ER Maastricht, The Netherlands; 7 Department of Pulmonary Disease, University Medical Center Groningen, University of Groningen, 9713 GZ Groningen, The Netherlands; 8 Department of Pulmonary Medicine, Erasmus MC, University Medical Center Rotterdam, 3015 CE Rotterdam, The Netherlands; 9 Institute for Diagnostic Accuracy, 9713 GH Groningen, The Netherlands; 10 Department of Respiratory Medicine, Amsterdam University Medical Center, 1081 HV Amsterdam, The Netherlands

**Keywords:** screening, lung cancer, selection criteria, risk prediction model, general population

## Abstract

**Background/Objectives**: The objective of this study is to evaluate the performance of the traditional age/smoking criteria and existing risk prediction models in selecting high-risk populations for lung cancer screening from a Western European general population. **Methods**: Baseline data from the Dutch population-based Lifelines cohort, collected between 2006 and 2013, were linked to the Dutch cancer registry to confirm lung cancer diagnoses. Five-year lung cancer risk was estimated based on traditional age/smoking criteria (NLST, NELSON, SPSTF-2021) and risk prediction models (LLPv2, PLCOm2012, Hoggart, Bach and Shanghai-LCM). For every strategy, the number of individuals eligible was determined, and total lung cancer cases in the eligible groups versus the ineligible groups were calculated. **Results**: Among 139,120 participants (aged ≥18 years), 218 (0.2%) developed lung cancer within five years. Age/smoking criteria identified 2161–6295 (1.6–4.5%) participants as eligible, comprising 62–92 (28.4–42.2%) lung cancer cases. Risk prediction models identified 2372–4315 (1.7–3.1%) participants as eligible, comprising 40–85 (18.4–38.9%) lung cancer cases. Among lung cancers in ineligible groups, 46.2–59.6% occurred in individuals who formerly smoked, and 28.7–39.3% occurred in individuals who currently smoke. Additionally, 41.2–70.0% of lung cancer cases in ineligible groups were in individuals younger than 50, and 44.3–72.3% in individuals who had quit smoking > 15 years prior to diagnosis. **Conclusions**: In a Western European population, current lung cancer screening selection criteria resulted in identifying only 18–42% of lung cancer cases. Cases in ineligible groups predominantly concern individuals who currently smoke and are below the threshold age and individuals who quit smoking > 15 years ago, highlighting the opportunity for more personalized risk-based screening strategies to increase lung cancer detection.

## Simple Summary

While much debate focuses on whether to implement lung cancer screening, a more fundamental question remains: who should be screened? Current selection criteria fail to optimally balance the trade-offs between resource allocation (cost, workforce capacity) and screening benefits (cancer detection, mortality reduction). Our findings emphasize the need to strongly improve selection criteria for lung cancer screening to maximize its benefit. To improve early cancer detection rates, we propose expanding current eligibility criteria to include: (1) individuals who currently smoke at younger ages and (2) individuals who formerly smoked with extended duration since smoking cessation.

## 1. Introduction

With almost 2.5 million new cases and over 1.8 million deaths, lung cancer is the leading cause of cancer morbidity and mortality worldwide [1]. Multiple randomized trials have demonstrated that low-dose computed tomography (LDCT) screening in high-risk populations reduces lung cancer mortality [2,3]. Currently, criteria for defining high-risk populations are primarily based on age and smoking history [2,3]. The updated US Preventive Services Task Force (USPSTF) guideline recommends annual LDCT in adults aged 50 to 80 with a smoking history of 20 pack-years or more, who currently smoke or have quit within the last 15 years [4]. In randomized controlled trials, lung cancer incidence rates of 5.58–6.45 cases per 1000 person-years have been reported [2,3].

To improve the efficacy (a higher number of cancers included per individual screened) of lung cancer screening, risk prediction models have been developed to identify individuals eligible for screening. These models incorporate multiple demographic, clinical and smoking-related factors, aiming to enhance the effectiveness and efficiency of LDCT screening programs by increasing the detection of lung cancer cases and/or reducing the number of individuals screened per lung cancer detected [5]. For example, the UK lung cancer screening program has integrated the Liverpool Lung Project version 2 (LLPv2) and the Prostate, Lung, Colorectal, and Ovarian Cancer Screening Trial 2012 (PLCOm2012) risk prediction models to select high-risk individuals. Eligibility includes individuals aged 55–74 years with an LLPv2 five-year risk of ≥2.5% or a PLCOm2012 six-year risk of ≥1.51% [6]. Studies have shown that risk prediction models outperform age- and smoking-based criteria in identifying high-risk individuals, demonstrating improved sensitivity and specificity [7].

However, despite incorporating additional risk factors beyond age and smoking history, a large number of lung cancer cases are still present in the ineligible groups who would not be invited to participate in screening. For instance, in a high-risk occupational cohort (New York City Fire Department rescue workers), the Bach and PLCOm2012 models failed to identify 49–54% of participants who developed lung cancer within 2001 to 2019 [8]. Most studies have evaluated the performance of risk prediction models within pre-selected, high-risk populations based on age and smoking criteria. The extent to which lung cancers occur in the general population outside age/smoking-based eligibility criteria, as well as the performance of risk prediction models in this broader context, remains unclear. This study, therefore, aims to quantify the number of lung cancer cases occurring among individuals deemed ineligible by age/smoking criteria and by externally validated existing risk prediction models in a Western European general population.

## 2. Materials and Methods

### 2.1. Study Design, Study Population

This study was conducted using data from Lifelines. Lifelines is a multi-disciplinary prospective population-based cohort study that uses a unique three-generation design to examine the health and health-related behaviors of 167,729 persons living in the north of the Netherlands. It employs a broad range of investigative procedures in assessing the biomedical, socio-demographic, behavioral, physical and psychological factors that contribute to the health and disease of the general population, with a special focus on multi-morbidity and complex genetics [9]. Baseline data were collected from over 167 thousand inhabitants between 2006 and 2013. For the presented analysis, only adult participants (≥18 years) were included. The Lifelines study received approval from the medical ethics review committee of the University Medical Center Groningen.

**Figure 1:**
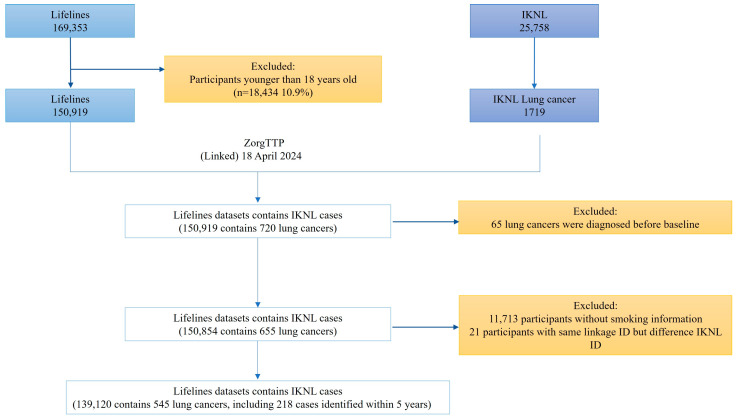
Flowchart of participants’ inclusion. IKNL: Netherlands Cancer Registry, Lifelines and IKNL were linked by last name, date of birth, gender, and initial.

Lifelines data were linked to the Netherlands Cancer Registry (NCR) to identify lung cancer cases. The NCR is a unique, population-based registry that contains individual patient data from all Dutch hospitals and radiotherapy institutes, providing comprehensive cancer statistics across the Netherlands [10]. Primary lung cancers were identified according to the 10th edition of the International Classification of Diseases (ICD-10). To ensure participant privacy, data linkage was performed by a trusted third party [11], using key identifiers including date of birth, gender, initials, and last name. The linked database included cancer diagnoses through December 2021. Lung cancers diagnosed prior to Lifelines’ baseline assessment were excluded. Further details on the linkage process are presented in Figure 1.

### 2.2. Risk Score Calculation

Eligibility for lung cancer screening was determined for Lifelines participants based on the National Lung Screening Trial (NLST), Nederlands–Leuvens Longkanker Screenings Onderzoek (NELSON) Trial, USPSTF criteria [2,3,4], and five existing risk prediction models: LLPv2 [12], PLCOm2012 [5], Shanghai lung cancer incidence model (Shanghai-LCM) [13], Hoggart model [14] and Bach model [15]. As the sojourn time for lung cancer to progress from the preclinical stage (detectable by screening test) to clinical stages is typically 3–6 years [16], a 5-year follow-up provides clinically relevant windows for risk prediction. Therefore, we used a 5-year timeframe to evaluate the performance of risk prediction models and traditional age/smoking criteria. For the risk score in the Bach and Hoggart models, we used the risk score calculations from the publicly available R package lcmodels [17]. Although the Bach model was developed for 10-year risk prediction, we rescaled it to a 5-year horizon for comparability with other risk prediction models, consistent with prior comparative studies [18]. For the Hoggart model, the risk score was computed as the sum over 5 years. For LLPv2 [12], Shanghai-LCM [13] and PLCOm2012 [5], risk estimates were calculated using published model coefficients, predictor definitions and age-standardized lung cancer incidence data. All models were applied using participant-level data according to the specifications described in the original publications [5,12,13,14,15]. Variables were summarized in Table S1.

### 2.3. Statistical Analysis

The participants were described both overall and stratified by the presence or absence of lung cancer diagnosed within 5 years of baseline assessment. Differences were assessed by Chi-square testing. Then, the models were described in terms of calibration and discrimination. Calibration was assessed by calculating the ratio of expected to observed number of lung cancer cases (E/O ratio) diagnosed within the 5-year follow-up. Generally, E/O ratios < 1 indicate underestimation of risk, while ratios > 1 indicate overestimation. Discrimination was evaluated using the area under the receiver operating characteristic curve (AUC).

We then assessed screening eligibility and potential lung cancer detection using the NLST, NELSON, USPSTF criteria [2,3,4] and risk prediction models. For the LLPv2 and PLCOm2012 models, we applied established UK screening thresholds (2.5% for LLPv2 and 1.51% for PLCOm2012 [6]). For the remaining models, thresholds were selected to match the proportion of participants deemed eligible by the LLPv2 model. For each method, the corresponding sensitivity, specificity, positive predicted value (PPV) and negative predicted value (NPV) were calculated.

The proportion of lung cancer deaths avoided for each age/smoking criteria and risk prediction models was estimated using cumulative rate ratios (CRRs) for lung cancer mortality in screening groups as reported in the NELSON study (0.76 for males and 0.670 for females) [2]. We assumed a 100% and 50% participation rate in a screening program, based on estimates from the NELSON and UK Lung Cancer Screening (UKLS) trials [2,19]. To better understand the limitations of current screening eligibility criteria and risk prediction models, we further characterized the individuals who developed lung cancer but were not eligible for screening under the different selection strategies, describing their age and smoking information. Finally, we specifically assessed these risk prediction models among individuals aged 55–74 with a history of smoking (both current and former), aligning with UK screening recommendations.

## 3. Results

### 3.1. Characteristics of the Study Population

Table 1 shows the baseline demographics and characteristics of the study participants. Among the 139,120 individuals aged ≥18 years in the Lifeline cohort, 218 individuals developed lung cancer (0.2%) within 5 years of follow-up. The most significant difference between individuals with and without lung cancer was the smoking status: 90.4% of individuals diagnosed with lung cancer were individuals who currently smoke or formerly smoked, while in the non-lung cancer groups, only 52.4%. Patients diagnosed with lung cancer exhibited heavier smoking behaviors compared to those without lung cancer: pack-years ≥ 30 with a smoking duration of ≥40 years versus pack-years < 10 with a duration of 10–19 years.

### 3.2. Calibration and Discrimination

**Figure 2:**
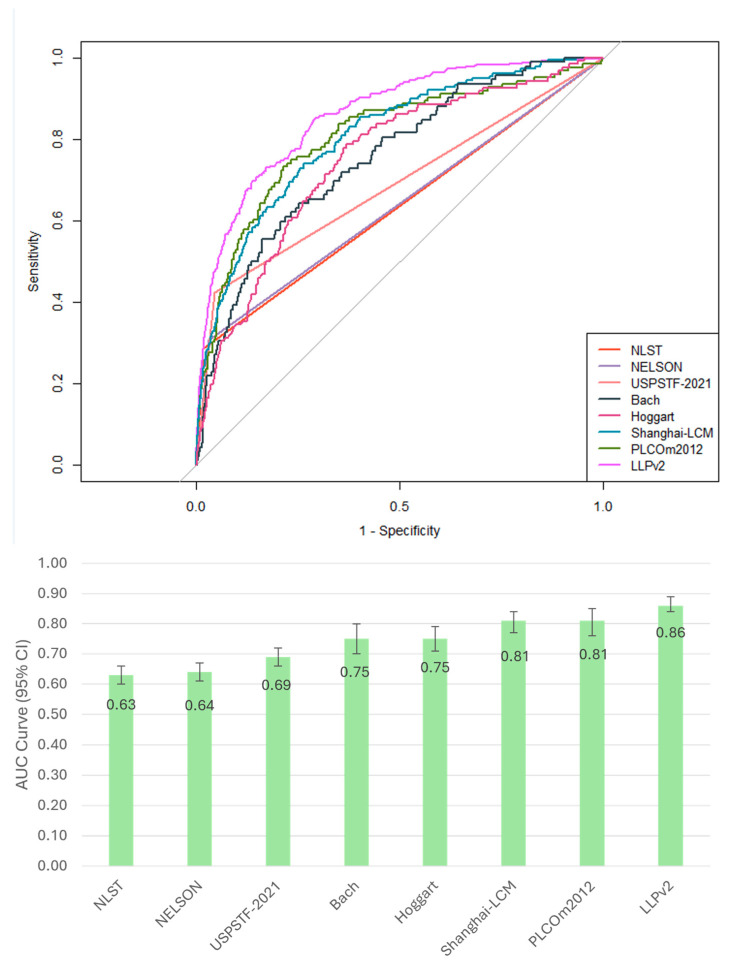
Discrimination of evaluated models and selection criteria. NLST: the National Lung Screening Trial; NELSON: the Dutch–Belgian Randomized Lung Cancer Screening Trial; USPSTF 2021: U.S. Preventive Services Task Force 2021 recommendation; LLPv2: the Liverpool Lung project Risk Model version 2; PLCOm2012: the Prostate, Lung, Colorectal and Ovarian Cancer Screening Trial Model 2012; Shanghai-LCM, Shanghai-Lung Cancer incidence Model; the AUC value of 0.50 indicates no discrimination (equivalent to random selection). Error bars represent 95% confidence intervals. AUC curve, area under the receiver operating characteristic curve.

Age/smoking selection criteria with E/O ratios ranged from 1.27 for the NLST criteria to 2.52 for the NELSON criteria. For risk prediction models, all overestimated the risk of lung cancer, with E/O ratios ranging from 1.02 to 7.20 (Figure S1). The highest AUC for lung cancer prediction was observed for LLPv2 (AUC 0.86), followed by PLCOm2012 and Shanghai-LCM (0.81), Hoggart and Bach (0.75), and USPSTF-2021 (0.69). Lower AUCs were observed for NELSON and NLST, with AUCs of 0.64 and 0.63, respectively (Figure 2).

### 3.3. Comparison of Age/Smoking Criteria and Risk Prediction Models for Lung Cancer Screening Eligibility

With the age/smoking criteria-based selection strategies of NLST, NELSON, and USPSTF-2021, 1.6–4.5% (2161–6295) of the participants would have been eligible for lung cancer screening. In the optimal situation of 100% participation and 100% detection, this would capture 28.4–42.2% (62–92) of lung cancers in these participants (Table 2). The PPVs ranged from 1.5 to 2.9%.

Using risk prediction models, 1.7–3.1% (2372–4315) of the participants would have been eligible for lung cancer screening, capturing 18.4–38.9% (40–85) of lung cancers. The best-performing models were LLPv2, Shanghai-LCM and PLCOm2012, which captured 85, 82 and 73 cancers (38.9%, 37.6% and 33.5%), yielding a PPV of 2.0%, 1.9% and 3.1%, respectively. The performance of risk prediction models in individuals aged 55–74 years who currently smoke or formerly smoked is presented in Table S2.

If all 218 lung cancers were captured in a screening program, 62 lung cancer deaths would be prevented (Table S4). With selection based on age/smoking criteria, 17 (26.8%) to 26 (41.1%) deaths from lung cancer would be prevented. With risk prediction models, 12 (19.3%) to 23 (37.3%) deaths would be prevented. With a 50% lower uptake, these numbers would be halved (Table S4).

### 3.4. Lung Cancers in the Ineligible Population

Cancer in individuals who have never smoked accounted for 9.6% of the lung cancers in this cohort (Table 3). These were not included in screening by all models or selection criteria. In the groups of ineligible individuals with lung cancer, 46.2–59.6% were diagnosed in individuals with a history of smoking, while 44.3–72.3% were not detected because these individuals quit smoking more than 15 years ago. If the threshold for quitting was shorter, 10 years (as in NELSON), an even higher proportion (77.3%) of cancers would not be included in this group. In individuals who currently smoke, 28.7–39.3% of cancer diagnoses would be ineligible for screening based on current criteria and risk models. Among individuals who currently smoke, when applying an age limit of 50 years, 41.2–70.0% of developing cancers would not be included, while applying an age limit of 55 years (as in NLST) would exclude 66.7% of individuals who currently smoke from screening eligibility. Lung cancers ineligible for screening among individuals aged 55–74 who currently or formerly smoke are provided in the supplementary materials (Table S3).

## 4. Discussion

In this study, we evaluated the performance of traditional age/smoking criteria and five known existing risk prediction models to identify participants eligible for lung cancer screening in a Western European general population. This study shows that the use of age/smoking criteria or one of the five risk prediction models in this population would mean 57.8–81.6% of lung cancers developing within 5 years would be in individuals who were not eligible for screening. Most of these individuals currently smoke and are younger than the age threshold or had previously smoked and had quit more than 15 years ago.

Risk prediction models outperform age/smoking criteria by including more lung cancer cases with comparable screening eligibility. In our study, the PLCOm2012 model with a 1.51% threshold selected a similar number of participants eligible for screening while including more cancers compared to the NLST criteria (73/2372 versus 62/2161). These findings are consistent with previous evidence, such as the Manchester Lung Health Check study, with the same threshold. PLCOm2012 selected 1429 participants with 62 cancers included, compared to 1188 individuals and 51 cancers identified under the NLST criteria [20]. For the Shanghai-LCM model [13], this study represents the first external validation. The model identified 37.6% of lung cancer cases, a performance comparable to that of the LLPv2 model (38.9%). Although the model was developed in an Asian population, the risk factors included are commonly used in lung cancer risk prediction models, such as smoking-related variables and family history of lung cancer, and do not include any Asian-specific risk factors.

In the ineligible groups, 44.3–77.3% of lung cancers were diagnosed in those who quit smoking over 10–15 years ago. The USPSTF recommends not to screen individuals who have not smoked for the last 15 years [4]. However, our findings reveal that individuals who formerly smoked and quit more than 15 years ago still face a significant risk of developing lung cancer, which is supported by other studies [21,22,23]. One study found that while the relative risk of lung cancer was halved after a median of 22 years since quitting, it remained substantially elevated, with a 10-fold higher risk compared to those who have never smoked [21]. Another study reported that individuals who quit smoking 15 to 30 years ago accounted for the largest percentage of lung cancer cases among those ineligible for screening [23]. Recently, the American Cancer Society updated its lung cancer screening guidelines, removing “years since quitting” as an eligibility criterion for individuals who formerly smoked [24]. National Health Interview Survey data reveal that among individuals with a considerable smoking history (≥20 pack-years), 50.5% quit ≥15 years. Landy et al. estimated that eliminating the criterion of years since quitting could result in an additional 8275 lung cancer deaths averted and 115,107 life years gained over five years in a population aged 50–80 years who currently smoke or formerly smoked [22].

Among individuals who currently smoke with lung cancers in the ineligible for screening groups, the majority (41.2–70.0%) were due to their exclusion based on the age threshold. Compared to the NLST criteria (aged 55–74 and at least 30 pack-years) [3], the USPSTF-2021 expanded the age range to 50–80 years and reduced the pack-year threshold to 20 [4]. However, 70.0% of lung cancers in those currently smoking in our study population occurred in individuals younger than 50 years at baseline (during a follow-up of 5 years). A population-level analysis in 110,000 participants from 26 lung cancer screening studies demonstrated that the proportion of stage I lung cancer cases decreases with age. These findings suggest that initiating screening at age 40 or 45 rather than at 50 or 55 could help detect early cancers that occur in younger individuals [25]. The detection of younger patients during screening, who subsequently receive incurable treatment, resulted in higher life years gained compared to older patients, thus improving the cost-effectiveness of the screening program.

Overall, 9.6% of the lung cancers occurred in individuals who had never smoked, and all these cancers were not eligible for screening. While this percentage is non-negligible, only 21 lung cancers were diagnosed among 66,244 adult never-smokers. According to the USPSTF, lung cancer screening is not recommended for individuals who have never smoked, as the potential harms of screening outweigh the conceivable benefits of detecting lung cancer early in this specific group [26]. In recent years, most screening trials involving individuals who have never smoked have been conducted in Asian countries where lung cancer incidence among this population is relatively high, largely due to other risk factors such as exposure to second-hand smoke, air pollution, cooking fumes and tuberculosis [27,28,29]. Despite promising detection rates (e.g., 2.6% in Taiwan) [27], challenges such as high false positives, over-diagnosis, and radiation-related risks remain, underscoring the need for improved risk stratification and personalized screening strategies for this specific population.

Our study has several strengths. First, the Lifelines cohort is a large, general population-based cohort, not selected by age and/or smoking status. It is broadly representative of socioeconomic characteristics, lifestyle, disease, and general health in the northern part of the Netherlands. Second, we provided a comprehensive and direct comparison of risk prediction models and selection criteria from current guidelines and two major screening trials. Therefore, our findings may be valuable for defining eligibility for lung cancer screening of individuals in the general population. Five existing lung cancer risk prediction models were assessed in this large population-based cohort, including those developed in the US, the UK, European countries, and China, all of which were created in regions with established lung cancer screening programs. By focusing on validated existing models rather than developing a new risk prediction model within this cohort, the study enables a robust comparison without the need for additional external validation or consideration of controlling confounders. Third, lung cancer cases were based on linkage with the NCR, a high-quality cancer registry with comprehensive nationwide coverage.

Our study also had some limitations. First, all five risk prediction models, as shown in Table 2, demonstrated some degree of risk overestimation in our cohort. Although the LLPv2 model has been updated to LLPv3, which shows less overestimation, we used LLPv2 because it is recommended by the NHS protocol for lung cancer screening. Such overestimation is common when risk prediction models are applied to external populations that differ from the development cohorts in terms of baseline risk and demographic characteristics. Second, we evaluated the performance of five risk prediction models in a single European country (the Netherlands) and did not account for known social, racial and geographic disparities in screening eligibility across countries. These disparities, which have been extensively discussed in the literature [30,31,32], may influence model performance and limit the generalizability of our findings to other populations. Third, smoking data in the Lifelines cohort are self-reported, which may introduce recall bias and potentially affect eligibility for lung cancer screening. However, observational studies suggest that retrospective recall of smoking history is generally reliable [33]. Fourth, the estimated proportion of potentially avoided deaths is based on prior trial data (NELSON) rather than on observed screening outcomes, introducing uncertainty in the estimated mortality benefit. Moreover, screening does not guarantee cancer detection [34], and mortality depends on multiple factors [35]. Fifth, the linkage between the IKNL and Lifelines cohorts was incomplete (42%), likely due to geographic differences, and may have introduced selection bias.

## 5. Conclusions

In conclusion, in this Western European population, a large percentage of lung cancers were diagnosed in individuals who are not currently eligible for lung cancer screening based on existing inclusion criteria. This shows the potential to capture a higher proportion of lung cancers if the inclusion criteria were broadened to include individuals who currently smoke at a younger age and individuals who formerly smoked with a longer duration since quitting. Furthermore, other data sources should be studied for the impact on balancing screening eligibility and lung cancer capture.

## 6. Patents

Patients were not involved in developing the research question, outcome measures, study design, or implementation, as identifying or engaging them was neither possible nor permitted.

## Figures and Tables

**Table 1:** Characteristics of adult participants in our cohort at baseline assessment (n = 139,120).

Variable	Total		Lung Cancer		No Lung Cancer		
	(n = 139,120)		(n = 218)		(n = 138,902)		*p*-Value
	No.	%	No.	%	No.	%	
Age							<0.001
<40	47,273	34.0%	<10	<3.2%	47,266	34.0%	
40–49	47,763	34.3%	41	18.8%	47,722	34.4%	
50–75	42,786	30.8%	155	71.1%	42,631	30.7%	
>75	1298	0.9%	15	6.9%	1283	0.9%	
Gender							<0.001
female	81,639	58.7%	102	46.8%	81,537	58.7%	
male	57,481	41.3%	116	53.2%	57,365	41.3%	
Smoking information							
Cigarette smoking status							<0.001
never	66,191	47.6%	21	9.6%	66,170	47.6%	
former	44,487	32.0%	109	50.0%	44,378	31.9%	
current	28,442	20.4%	88	40.4%	28,354	20.5%	
Age at starting smoking (current and former smokers)							<0.001
<10	175	0.2%	<10	<1.5%	172	0.2%	
10–19	63,768	87.4%	180	91.4%	63,588	87.5%	
20–29	7680	10.5%	13	6.6%	7667	10.5%	
30–39	918	1.3%	<10	<0.5%	917	1.3%	
≥40	388	0.5%	0	0.0%	388	0.5%	
Cigarettes per day (current and former smokers)							<0.001
<10	31,375	43.0%	37	18.8%	31,338	43.1%	
10–19	31,082	42.6%	103	52.3%	30,979	42.6%	
20–29	9153	12.6%	43	21.8%	9110	12.5%	
≥30	1319	1.8%	14	7.1%	1305	1.8%	
Pack-years (current and former smokers)							<0.001
<10	40,362	55.3%	26	13.2%	40,336	55.5%	
10–19	19,326	26.5%	39	19.8%	19,287	26.5%	
20–29	8322	11.4%	42	21.3%	8280	11.4%	
≥30	4919	6.7%	90	45.7%	4829	6.6%	
Smoking duration (current and former smokers)							<0.001
<10	17,156	23.5%	<10	<3.0%	17,150	23.6%	
10–19	24,126	33.1%	18	9.1%	24,108	33.1%	
20–29	17,152	23.5%	38	19.3%	17,114	23.5%	
30–39	10,639	14.6%	54	27.4%	10,585	14.6%	
≥40	3856	5.3%	81	41.1%	3775	5.2%	

Note: Data are presented as numbers (percentages); Chi-square test for categorical variables.

**Table 2:** Implication of age/smoking criteria and risk prediction models for participant selection.

Selection Tools	NLST	NELSON	USPSTF-2021	LLPv2	PLCOm2012	Shanghai-LCM	Bach	Hoggart
Participants	2161	4357	6295	4315	2372	4278	4114	4149
LCs in eligible groups	62 (28.4%)	67 (30.7%)	92 (42.2%)	85 (38.9%)	73 (33.5%)	82 (37.6%)	72 (33.0%)	40 (18.4%)
LCs in ineligible groups	156	151	126	133	145	136	146	178
Sensitivity	28.4%	30.7%	42.2%	39.0%	33.5%	37.6%	33.0%	18.3%
Specificity	98.5%	96.9%	95.5%	97.0%	98.3%	97.0%	97.1%	97.0%
PPV	2.9%	1.5%	1.5%	2.0%	3.1%	1.9%	1.8%	1.0%
NPV	99.9%	99.9%	99.9%	99.9%	99.9%	99.9%	99.9%	99.9%
Model criteria	Aged 55–75, ≥30 pack-years, or quit ≤ 15 years	Aged 50–75, ≥15 cigarettes per day for ≥25 years, or quit ≤ 10 years	Aged 50–80, ≥20 pack-years, or quit ≤ 15 years	Aged 20–80 all smokers with a model threshold of 2.50%	Aged 55–74 ever-smokers with a model threshold of 1.51%	Aged 40–75 ever-smokers with a model threshold of 1.00%	Aged 45–69, ≥20 pack-years, or quit ≤ 15 years with a model threshold of 0.8%	Aged 40–65 ever-smokers with a model threshold of 5.6%

Note: LCs, lung cancer cases; NLST: the National Lung Screening Trial; NELSON: the Dutch–Belgian Randomized Lung Cancer Screening Trial; USPSTF-2021: U.S. Preventive Services Task Force 2021 recommendation; LLPv2: the Liverpool Lung project Risk Model version 2; PLCOm2012: the Prostate, Lung, Colorectal and Ovarian Cancer Screening Trial Model 2012, risk score calculated for 6-years; Shanghai-LCM, Shanghai-Lung Cancer incidence Model; PPV: positive predictive value; NPV: negative predictive value.

**Table 3:** Age and smoking information for lung cancers in ineligible groups.

Selection Tools	NLST (n = 156)	NELSON (n = 151)	USPSTF-2021 (n = 126)	LLPv2 (n = 133)	PLCOm2012 (n = 145)	Shanghai-LCM (n = 136)	Bach (n = 146)	Hoggart (n = 178)
Age under cutoff	44.2% < 55 yrs (69/156)	31.8% <50 yrs (48/151)	38.1% < 50 yrs (48/126)	36.1% < 50 yrs (48/133)	33.1% < 50 yrs (48/145)	35.3% < 50 yrs (48/136)	30.8% < 50 yrs (45/146)	23.0% < 50 yrs (41/178)
Age above cutoff	10.3% > 75 yrs (16/156)	9.9% > 75 yrs (15/151)	3.2% > 80 yrs (4/126)	3.0% > 80 yrs (4/133)	2.8% > 80 yrs (4/145)	2.9% > 80 yrs (4/136)	2.7% > 80 yrs (4/146)	2.2% > 80 yrs (4/179)
Individuals who formerly smoked (< age cutoff)	29.5% < 55 yrs (23/78)	17.3% < 50 yrs (13/75)	20% < 50 yrs (13/65)	21.0% < 50 yrs (13/62)	19.4% < 50 yrs (13/67)	18.3% < 50 yrs (13/71)	16.9% < 50 yrs (13/77)	12.3% < 50 yrs, (13/106)
Individuals who currently smoke (< age cutoff)	66.7% < 55 yrs (38/57)	50.9% < 50 yrs (28/55)	70.0% < 50 yrs (28/40)	56.0% < 50 yrs (28/50)	49.1% < 50 yrs (28/57)	63.6% < 50 yrs (28/44)	52.1% < 50 yrs (25/48)	41.2% < 50 yrs (21/51)
Individuals who have never smoked (< age cutoff)	38.1% < 55 yrs (8/21)	33.3% < 50 yrs (7/21)	33.3% < 50 yrs (7/21)	33.3% < 50 yrs (7/21)	33.3% < 50 yrs (7/21)	33.3% < 50 yrs (7/21)	33.3% < 50 yrs (7/21)	33.3% < 50 yrs (7/21)
smoking duration beyond cutoff	-	27.7% < 25 smy; (36/130); 47.7% < 30 smy; (62/130)	-	-	-	-	-	-
cigarettes per day beyond cutoff	-	56.9% < 15 cpd (74/130); 28.5% < 10 cpd (37/130)	-	-	-	-	-	-
pack-years beyond cutoff	79.3% < 30 py (107/135)	-	61.9% < 20 py (65/105)	46.4% < 20 py (52/112)	48.4% < 20 py (60/124)	49.6% < 20 py (57/115)	52.0% < 20 py (65/125)	38.9% < 20 py (61/157)
quit years	19.0 ± 12.1	20.2 ± 11.4	23.7 ± 11.1	17.5 ± 12.3	18.9 ± 12.7	18.9 ± 12.5	19.4 ± 11.9	15.5 ± 12.2
quit years beyond cutoff	60.3% > 15 qy (47/78)	77.3% > 10 qy (58/75)	72.3% > 15 qy (47/65)	61.3% > 15 qy (31/62)	58.2% > 15 qy (39/67)	57.7% > 15 qy (41/71)	61.0% > 15 qy (47/77)	44.3% > 15 qy (47/106)

Note: yrs = years; py = pack-years; qy = quit years; cpd = cigarettes per day; smy = smoking years. For age/smoking criteria, cutoffs for age and smoking information was based on its own cutoffs; NLST: age < 55 years, <30 pack years, or individuals who formerly smoked with >15 quit years; NELSON, age < 50 years, <15 cigarettes per day, <25 years of smoking duration; <15 cigarettes per day, <10 years of smoking duration, and individuals who formerly smoked with >15 quit years; USPSTF: age <50 years, individuals who currently smoke with <20 pack-years, and individuals who formerly smoked with >15 quit years. For risk prediction models, the USPSTF-2021 guidelines were applied to assess age and smoking information among missed cancers across all models.

## Data Availability

Data may be obtained from a third party and are not publicly available. Researchers can apply to use the Lifelines data used in this study. More information about how to request Lifelines data and the conditions of use can be found on their website (https://www.lifelines-biobank.com/researchers/working-with-us, accessed on 12 October 2023).

## Supplementary Materials

The following supporting information can be downloaded at: https://www.mdpi.com/article/10.3390/cancers18050724/s1, Figure S1: Calibration of evaluated age/smoking criteria and risk models based on the selection strategies’ criteria; Table S1: Summary of risk prediction models; Table S2: Performance of risk-prediction models on 55–74 ever smokers; Table S3: Lung cancers in ineligible groups for standardized population; Table S4: Rough estimation on lung cancer deaths avoided at 10 years based on the selection strategies’ criteria.

## Abbreviations

The following abbreviations are used in this manuscript:

AUC	Area under the curve
CRRs	Cumulative risk ratios
E/O ratios	Expected/observed ratios
LDCT	Low-dose computed tomography
LLPv2	Liverpool Lung Project version 2
NELSON	Nederlands–Leuvens Longkanker Screenings Onderzoek
NLST	National Lung Screening Trial
NPV	Negative predictive value
PLCOm2012	Prostate, Lung, Colorectal, and Ovarian Cancer Screening Trial 2012
PPV	Positive predicted value
Shanghai-LCM	Shanghai lung cancer incidence model
USPSTF-2021	US Preventive Services Task Force
