# Supplementary material for: Improving Lung Cancer Screening Selection: A Comparative Analysis of Risk Models and Traditional Criteria in a Western European General Population
Source: Cancers (Basel). 2026 Feb 24;18(5):724. doi: 10.3390/cancers18050724 (PMC12984101; doi:10.3390/cancers18050724)
Supplement: Supplementary file 1 [file cancers-18-00724-s001.zip › cancers-4123273-supplementary.pdf]

Age/smoking-based selection and risk-prediction models

We evaluated selection criteria from NLST and NELSON, the two largest randomized-controlled screening trials, the 2021 USPSTF selection criteria and five lung cancer risk-prediction models, to determine their performance in identifying individuals eligible for screening and the number of lung cancer cases detected.

The first two risk-prediction models, the Bach model [1], and the Prostate, Lung, Colorectal and Ovarian Cancer Screening Trial Model 2012 (PLCOm2012) [2], were developed within US populations. The Shanghai lung cancer incidence model (Shanghai-LCM) [3], was developed in a Chinese cohort and validated in Asian cohorts. The final two models, the Liverpool Lung project Risk Model version 2 (LLPv2) [4], and the Hoggart model [5], were developed by UK research teams. The Hoggart model was developed based on Western European cohorts, including populations from Italy, Spain, England, the Netherlands, Greece, Germany, Denmark and Norway. Details of the included criteria and risk-prediction models are provided in Table S1.

Table S1 Summary of risk prediction models

Models	Cohort used for development	Pre-selection criteria	Risk factors in the model
LLPv2<br>[4]	Liverpool Lung Project<br>Lung cancer case-control cohort	Aged 20-80 years	Age, gender, COPD/emphysema/Bronchitis, pneumonia, previous cancer, smoking duration, family lung cancer history (early-onset, late-onset), asbestos exposure.
Hoggart<br>[5]	European Prospective Investigation into Cancer and Nutrition (EPIC) cohort<br>Pre-selected cohort from the general population	Aged 40-65 years, ever smokers	Smoking status, smoking duration, starting age, cigarettes per day.
PLCOm2012<br>[2]	Prostate, Lung, Colorectal and Ovarian Cancer Screening Trial.<br>Cohort from screening randomized clinical trial	Aged 55-75 years, ever smokers	Age, race, education, BMI, previous cancer, COPD, family history of lung cancer, smoking status, smoking duration, cigarettes per day, years since cessation.
Bach<br>[1]	Carotene and Retinol Efficacy Trial (CARET)<br>Cohort from lung cancer prevention trial	Aged 45-69 years, ever smokers,≥20 pack-years history, or quit≤15 years	Age, gender, smoking duration, cigarettes per day, years since cessation, asbestos exposure.
Shanghai-LCM<br>[3]	Shanghai Men's Health Study;<br>Shanghai Women's Health Study<br>Pre-selected ever-smokers from the general population	Aged 40-75 years, ever smokers	Age, gender, education, BMI, smoking duration, pack-years, number of lung cancers in family members.

Abbreviations: LLPv2, Liverpool Lung Project Incidence Risk Model version2; Hoggart, the Hoggart Model; PLCOm2012, Prostate, Lung, Colorectal, and Ovarian Cancer Screening Trial Model 2012; Bach, Bach Model; Shanghai-LCM, Shanghai lung cancer incidence model.

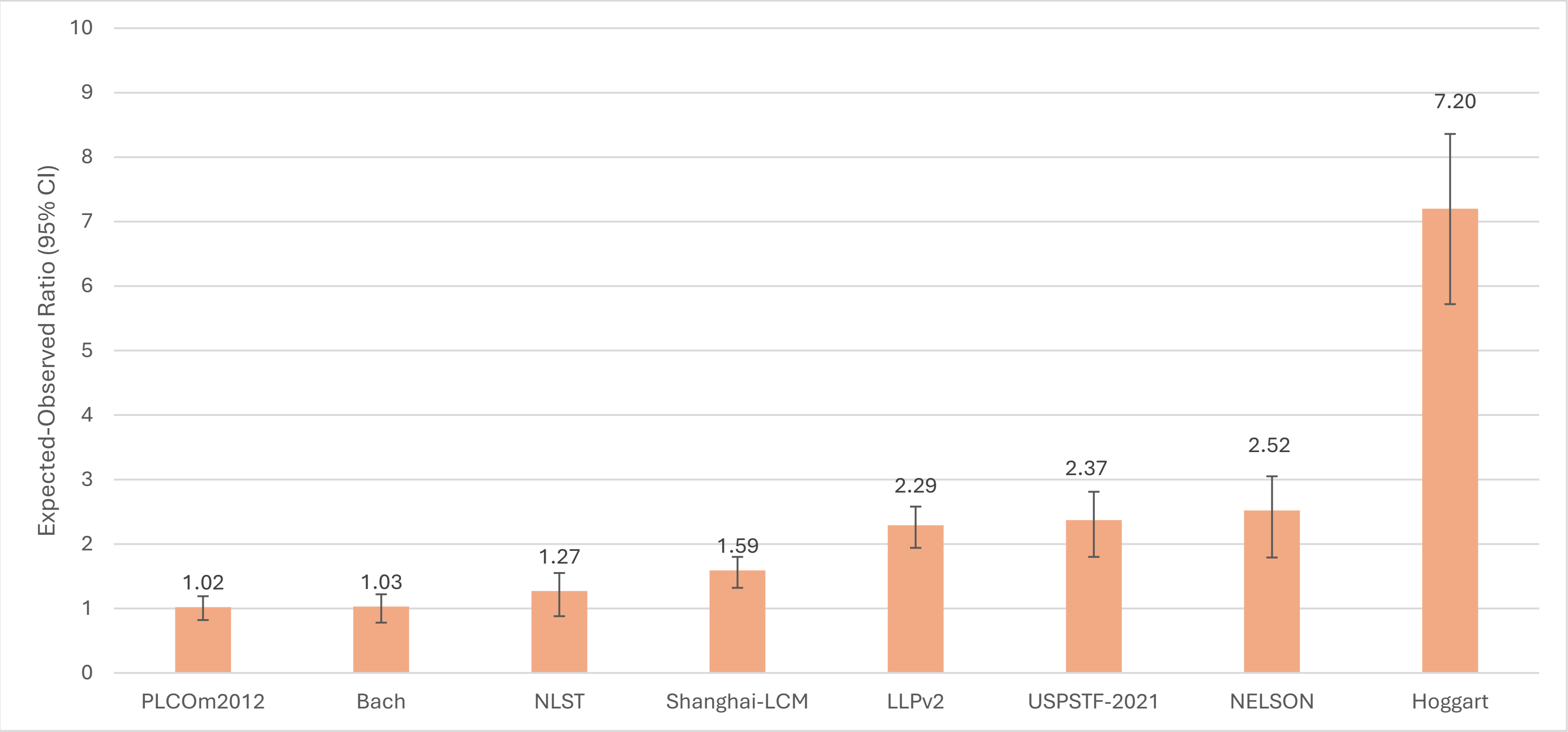

Figure S1 Calibration of evaluated age/smoking criteria and risk models based on the selection strategies' criteria

Expected-observed ratios (E/O) are the mean value across quintile. Risk score sorted ascendingly and then split into quintiles, where in each quintile the expected number of lung cancers was calculated by summing the predicted risk for all individuals in that quintile. For each screening strategy (NLST, NELSON, and USPSTF-2021), participants in the present cohort who met the corresponding eligibility criteria were identified. The expected number of lung cancer cases was calculated by multiplying the number of eligible participants by the lung cancer incidence proportion reported in the original trial or guideline-defining population for each strategy. The observed number of lung cancer cases was defined as the number of cancers diagnosed among eligible participants during follow-up in the present cohort. The E/O ratio was calculated as the ratio of expected to observed cases. E/O less than 1 indicate

underestimation of the risk and those greater than 1 indicate overestimation of the risk. The Hoggart model substantially overestimated lung cancer risk in the Lifelines cohort, this overestimation is likely attributable to differences in baseline lung cancer risk and population characteristics between the model development and the general population evaluated in this study. In particular, the Hoggart model was developed using participants recruited between 1993 and 1999 across Western Europe, when smoking prevalence and smoking intensity were substantially higher.

Table S2 Performance of risk-prediction models on 55-74 ever smokers

Risk prediction models	LLPv2	PLCOm2012	Shanghai-LCM	Bach	Hoggart
**Participants**	3836	2372	3953	3661	3568
**LCs captured**	76	73	79	83	73
**sensitivity**	61.8%	59.3%	64.2%	67.5%	59.3%
**specificity**	78.4%	86.8%	77.8%	79.5%	79.9%
**PPV**	2.0%	3.1%	2.0%	2.3%	2.0%
**NPV**	99.66%	99.67%	99.68%	99.71%	99.64%
**thresholds**	2.50%	1.51%	1.00%	1.20%	3.85%

Note: only thresholds were applied in standardized population (55-74 ever smokers), with 123 lung cancers in 17,546 participants in this standardized population.

Table S3 Lung cancers in ineligible groups for standardized population

**Lung cancers in ineligible groups outside of 55-74 ever smokers (n=95)**					
Never smokers (21)	<55yrs: 33.3% (7/21);				
Individuals who currently smoke (40)	<55yrs: 92.5% (37/40);<br><55yrs and >20pys: 62.2% (23/37)				
Individuals who formerly smoked (34)	<55yrs: 67.6% (23/34);<br><55yrs and >20pys: 30.4%(7/23)				
**Lung cancers ineligible for screening within individuals aged 55-74 with smoking history (both current and former)**					
**Risk prediction models**	**LLPv2 (n=47)**	**PLCOm2012 (n=50)**	**Shanghai-LCM(n=44)**	**Bach (n=40)**	**Hoggart (n=50)**
Individuals who currently smoke	N=11<br>Age: 59.1±2.6<br>Packyeaers:28.7±2.6	N=17<br>Age:60.5±2.9<br>Packyears:25.8±1.7	N=6<br>Age:59.8±3.3<br>Packyears:27.4±8.4	N=7<br>Age:58.9±3.2<br>Packyears:23.0±10.7	N=9<br>Age:60.6±3.0<br>Packyears:28.5±15.5

Individuals who formerly smoked	N=36<br>Age:67.7±4.9<br>Packyears:23.4±17.4<br>Quit years:21.7±12.1	N=33<br>Age:62.8±5.2<br>Packyears:18.5±15.2<br>Quit years:23.2±12.4	N=38<br>Age:63.8±5.6<br>Packyears:20.7±16.3<br>Quit years:22.7±11.9	N=33<br>Age:63.1±5.3<br>Packyears:16.1+11.1<br>Quit years:22.9±12.9	N=41<br>Age:63.1±5.3<br>Packyears:23.8±8.4<br>Quit years:21.1±12.9

Table S4 Rough estimation on lung cancer deaths avoided at 10 years based on the selection strategies' criteria

**Age/smoking criteria and models**	**NLST**	**NELSON**	**USPSTF-2021**	**LLPv2**	**PLCOm2012**	**Shanghai-LCM**	**Bach**	**Hoggart**
**Proportion of lung cancer deaths avoided by different tools***<br>**(100% response uptake)**	**26.8% (17/62)**	**30.0% (19/62)**	**41.1% (26/62)**	**37.3% (23/62)**	**36.5% (20/62)**	**36.5% (23/62)**	**32.4% (20/62)**	**19.3% (12/62)**
**Proportion of lung cancer deaths avoided by different tools #**<br>**(50% response uptake)**	**13.4% (8/62)**	**15.0% (9/62)**	**20.6% (13/62)**	**18.6% (12/62)**	**16.3% (11/62)**	**18.3% (11/62)**	**16.2% (10/62)**	**9.6% (6/62)**

Note: If all 218 lung cancers were captured (116 males and 102 females), then based on the cumulative rate ratio of lung cancer mortality at 10 years for males and females from the NELSON study (0.76 for male and 0.67 for female), the number of avoided deaths from lung cancer deaths would total 62.

* Estimated based on the age/smoking criteria or model's threshold.

# Estimated based on age/smoking criteria or model's threshold, combined with 50% response uptake.

Reference:

1. Bach PB, Kattan MW, Thornquist MD, et al. Variations in lung cancer risk among smokers. J Natl Cancer Inst. 2003;95(6):470-8. doi:10.1093/jnci/95.6.470
2. Tammemagi MC, Katki HA, Hocking WG, et al. Selection criteria for lung-cancer screening. N Engl J Med. 2013;368(8):728-36. doi:10.1056/NEJMoa1211776
3. Yang JJ, Wen W, Zahed H, et al. Lung Cancer Risk Prediction Models for Asian Ever-Smokers. J Thorac Oncol. 2024;19(3):451-64. doi:10.1016/j.jtho.2023.11.002
4. Cassidy A, Myles JP, van Tongeren M, et al. The LLP risk model: an individual risk prediction model for lung cancer. Br J Cancer. 2008;98(2):270-6. doi:10.1038/sj.bjc.6604158
5. Hoggart C, Brennan P, Tjonneland A, et al. A risk model for lung cancer incidence. Cancer Prev Res (Phila). 2012;5(6):834-46. doi:10.1158/1940-6207.CAPR-11-0237
